# Multiple shoot induction from axillary bud cultures of the medicinal orchid, *Dendrobium longicornu*

**DOI:** 10.1093/aobpla/pls032

**Published:** 2012-11-05

**Authors:** Stadwelson Dohling, Suman Kumaria, Pramod Tandon

**Affiliations:** 1 Department of Botany, Lady Keane College, Shillong, Meghalaya 793 001, India; 2 Plant Biotechnology Laboratory, Department of Botany, North-Eastern Hill University, Shillong, Meghalaya 793 022, India

## Abstract

**Background and aims:**

*Dendrobium longicornu*, commonly known as the ‘Long-horned *Dendrobium*’, is an endangered and medicinally important epiphytic orchid. Over-exploitation and habitat destruction seriously threaten this orchid in Northeast India. Our objective was to develop an efficient protocol for the mass propagation of *D. longicornu* using axillary bud segments.

**Methodology and principal results:**

Axillary buds cultured in Murashige and Skoog semi-solid medium supplemented with α-naphthalene acetic acid (NAA), 2,4-dichlorophenoxy acetic acid (2,4-D) and 6-benzylaminopurine (BAP) readily developed into plantlets. These formed either directly from shoot buds or from intermediary protocorm-like bodies (PLBs). The maximum explant response (86.6 %) was obtained in medium supplemented with NAA at 30 µM, while the maximum number of shoots (4.42) and maximum bud-forming capacity (3.51) were observed in medium containing 15 µM BAP and 5 µM NAA in combination. Protocorm-like bodies were obtained when the medium contained 2,4-D. The maximum number of explants forming PLBs (41.48 %) was obtained in medium containing 15 µM BAP and 15 µM 2,4-D. Well-developed plantlets obtained after 20–25 weeks of culture were acclimatized and eventually transferred to the greenhouse. Over 60 % of these survived to form plants ∼3–4 cm tall after 90 days in glasshouse conditions using a substrate of crushed brick and charcoal, shredded bark and moss.

**Conclusions:**

The method described can readily be used for the rapid and large-scale regeneration of *D. longicornu*. Its commercial adoption would reduce the collection of this medicinally important and increasingly rare orchid from the wild.

## Abstract (precis)

The present investigation was undertaken to propagate *D. longicornu*, a medicinally important orchid using axillary bud segments. This approach could also help in conserving other threatened orchids as well.

## Introduction

**Fig. 1:**
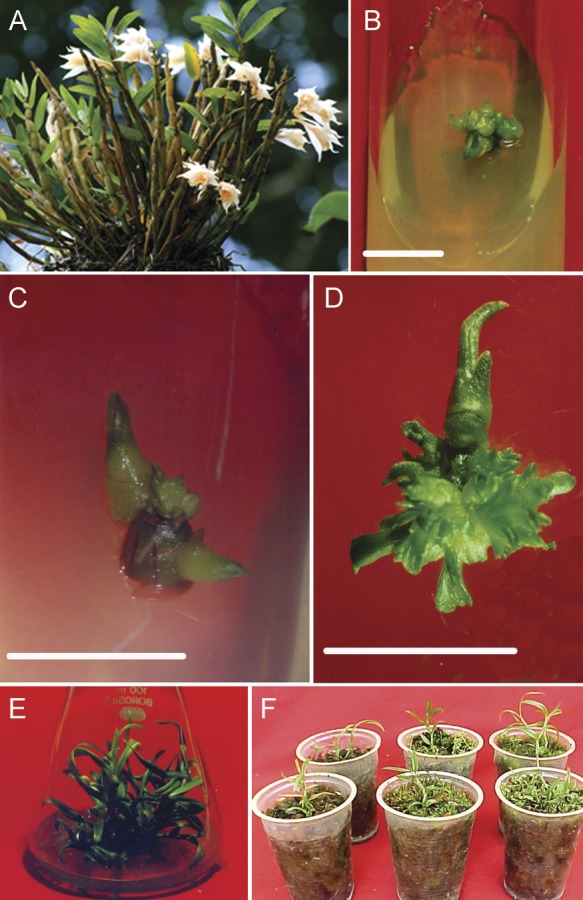
***In vitro* propagation of *D. longicornu* from 3- to 4-mm- long nodal explants.** (A) Mature plant in the natural habitat. (B) Initiation of PLBs on explants cultured on MS medium containing 15 µM BAP and 15 µM 2,4-D for 15 days (bar = 1 cm). (C) Initiation of shoots from an axillary bud on medium containing 5 µM BAP and 15 µM NAA in 15 days (bar = 1 cm). (D) As in (C) but after 30 days (bar = 1 cm). (E) Rooted plantlets after 70 days in culture. (F) Hardened plantlets after 90 days.

*Dendrobium longicornu* is an endemic orchid of Northeast India (Chowdhery 2001) (Fig. 1A). It is medicinally important and extracts are used to treat fever and coughs (Manandhar 1995). This orchid is included in the Convention of International Trade for Endangered Species (CITES) list (**www.cites.org/eng/cop/12/doc/E 12–64.pdf**). Collection from the wild and extensive and continuing habitat destruction due to deforestation and other unplanned human activities are proceeding at a pace, and are depleting the natural populations of orchids. A method of fast mass propagation is urgently needed. Up until now, clonal propagation has been mainly through the separation of root-bearing adventitious growths (keikis) from the mother stem. However, this is slow and unsuitable for mass propagation. Tissue culture is an alternative approach and various methods have already been developed to promote multiplication of *Dendrobium* (Arditti and Ernst 1993; Kumaria and Tandon 2001; Dohling *et al.* 2007; Zhao *et al.* 2007; Luo *et al.* 2008; Chugh *et al.* 2009; Paul *et al*. 2012). However, the technique needed for success differs between species. It has also been reported that explant response to tissue culture varies with their source, physiological state and nutrient environment (Vij *et al*. 1983). Information on how best to micropropagate *D. longicornu* is lacking (Dohling *et al.* 2008). In this paper, we rectify this shortcoming by reporting a rapid method for its large-scale multiplication.

## Methods

Nodal explants of *D. longicornu* were obtained from plants maintained in the net house of the Plant Biotechnology Laboratory, Department of Botany, North-Eastern Hill University, Shillong, India. Stem explants (∼1–2 cm long), each comprising a node and axillary bud, were cleaned by gently scrubbing with a soft brush and mild detergent, washed in running tap water for 15–20 min and rinsed with distilled water. The explants were surface sterilized with 10 % (v/v) NaClO solution (4–6 % available chlorine; Merck) for 10 min followed by 0.1 % (w/v) HgCl_2_ (HiMedia) for 2 min. After washing 5–6 times with sterilized distilled water, the explants were shortened to 3–4 mm after the removal of leaves, dry sheaths and other external tissues. These were then cultured in Murashige and Skoog (MS) medium (Murashige and Skoog 1962) containing 3 % sucrose (HiMedia), 0.8 % agar (HiMedia) and supplemented with the growth regulators α-naphthalene acetic acid (NAA), 2,4-dichlorophenoxy acetic acid (2,4-D) and 6-benzyl-aminopurine (BAP) using a range of concentrations between 0 and 50 µM, and in various combinations. The aim was to optimize the formation of protocorm-like bodies (PLBs) and shoot buds in the cultured explants. The cultures were incubated at 25 ± 2 °C under a 12-h photoperiod of 50 μmol m^−2^ s^−1^ photon flux density. There were 10 replicates of each treatment and all experiments were repeated three times. Observations were made after 45 days of culture. Well-developed plantlets with roots were obtained in the same induction medium on being left undisturbed. After 20–25 weeks of culture these plantlets were transferred to perforated ‘Thermocol’ or propylene plastic pots (10 × 7 cm size) containing a range of composts: (i) crushed brick and charcoal (1 : 1); (ii) crushed brick and charcoal (1 : 1) + a top layer of moss; (iii) crushed brick and charcoal, and decaying litter (1 : 1 : 1); (iv) crushed brick and charcoal, and decaying litter (1 : 1 : 1) + a top layer of moss; (v) crushed brick and charcoal, and shredded bark (1 : 1 : 1); (vi) crushed brick and charcoal chunks, and shredded bark (1 : 1 : 1) + a top layer of moss. Sixty plantlets were grown in each of these composts in the greenhouse, covered with a pierced polythene bag for 3 weeks, and sprayed with water to inhibit dehydration. The temperature was maintained at 24 ± 2 °C, and relative humidity at 70–80 %. Plantlets were irrigated with 1/10 strength MS medium solution every 2 days for 2 weeks. Plants were assessed after 90 days.

The data were subjected to statistical analysis using one-way analysis of variance and comparisons between the mean values of treatments were made by Fisher's least significant difference (LSD) test (Fisher 1935). For a more realistic determination of the efficacy of a given treatment, regeneration of shoot buds from axillary buds was calculated as the bud-forming capacity (BFC) using the formula (Tandon *et al*. 2007): BFC = (average number of buds per explant × % of explants forming buds)/100.

## Results

Two auxins (NAA and 2,4-D) and a cytokinin (BAP) were tested separately, each at four concentrations (5, 15, 30 and 50 µM). After 45 days of culture, nodal explants each with an axillary bud produced the most shoots in MS media containing NAA, but no PLBs were observed. The highest response (86.6 %) with the maximum number of shoots (3.28) and a BFC of 2.84 per explant was obtained using 30 µM NAA (Table 1). Explants cultured in media containing 2,4-D rather than NAA gave a more variable response by forming both shoots and PLBs with maximum PLB production occurring at a concentration (30 µM) that inhibited shoot production completely. Explants treated with BAP but no auxin produced no PLBs and with shoot production and bud-forming capacity somewhat over half of that seen in NAA (Table 1).

**Table 1:** Effect of growth regulators incorporated singly in MS medium on formation of shoot buds and PLBs.

Concentration (μM)	Explant response (%)	Average number of shoots per explant*	Bud-forming capacity	Explant response into PLBs (%)
Control	70.0 ± 5.7	1.71 ± 0.06^c^	1.20	–
NAA				
5	70.0 ± 2.1	2.10 ± 0.05^b^	1.47	–
15	70.0 ± 3.1	2.23 ± 0.26^b^	1.56	–
30	86.6 ± 3.3	3.28 ± 0.28^a^	2.84	–
50	40.0 ± 4.1	1.55 ± 0.11^c^	0.62	–
2, 4–D				
5	66.4 ± 2.1	1.38 ± 0.19^cd^	0.92	29.07 ± 5.5
15	65.4 ± 2.2	1.36 ± 0.07^cd^	0.89	28.51 ± 3.2
30	36.9 ± 1.9	–		36.9 ± 1.9
50	–	–		–
BAP				
5	60.0 ± 0.0	1.44 ± 0.05^cd^	0.86	–
15	63.3 ± 2.3	1.61 ± 0.14^c^	1.02	–
30	36.6 ± 3.3	1.41 ± 0.21^cd^	0.52	–
50	30.0 ± 0.0	1.00 ± 0.00^d^	0.30	–

*Values are the mean ± SE. Means followed by the same letter in the column are not significantly different as indicated by Fisher's LSD (*P* = 0.05).

In further tests, each auxin at 5, 15, 30 or 50 µM was combined with BAP at concentrations of 5, 15 or 30 µM (Table 2). As before, media containing NAA formed the most shoots directly, with a combination of 15 µM BAP and 5 or 15 µM NAA being the most effective. Remarkably, at 15 µM NAA and 15 µM BAP, large numbers of PLBs were also formed. Generally, explants in media containing 2,4-D again formed mostly PLBs, but with some direct production of shoots as well. However, when 15 or 30 µM 2,4-D was combined with BAP at 15 µM, almost all direct shoot production was channelled into PLB formation. The highest explant response (72.59 %) and PLB conversion (41.48 %) were observed at 15 µM each of BAP and 2,4-D together (Table 2; Fig. 1B). The response of axillary buds was, however, significantly low as compared with the control in the medium with high concentrations of BAP. A high response (81.2 %) of the explants was recorded in the medium containing 15 µM BAP and 5 µM NAA in combination, while an increased number of shoots (4.42 per explant) and the highest BFC of 3.51 were recorded with the treatment containing 5 µM BAP and 15 µM NAA (Table 2; Fig. 1C and D).

**Table 2:** Effect of growth regulators in combination in MS medium on formation of shoot buds and PLBs.

Treatments (μM)		Explant response (%)	Average number of shoots per explant*	Bud-forming capacity	Explant response into PLBs (%)
Control		70.0 ± 5.7	1.71 ± 0.06^e^	1.20	–
BAP	NAA				
5	5	75.5 ± 2.4	2.15 ± 0.17^d^	1.62	–
5	15	79.3 ± 0.7	4.42 ± 0.24^a^	3.51	–
5	30	57.1 ± 1.4	2.22 ± 0. 11^d^	1.27	–
5	50	56.1 ± 1.1	1.75 ± 0.04^e^	0.98	–
15	5	81.2 ± 2.3	3.20 ± 0.10^b^	2.60	–
15	15	80.8 ± 3.6	2.85 ± 0.14^bc^	2.30	38.75 ± 0.72
15	30	59.7 ± 3.4	2.27 ± 0.11^d^	1.35	–
30	5	56.6 ± 3.3	2.85 ± 0.26^bc^	1.61	–
30	15	62.2 ± 2.2	2.61 ± 0.14^cd^	1.62	–
30	30	53.3 ± 3.3	2.43 ± 0.11^cd^	1.30	22.40 ± 1.4
					
BAP	2,4-D				
5	5	65.55 ± 2.9	1.55 ± 0.05^e^	1.02	31.11 ± 1.1
5	15	63.33 ± 3.3	1.47 ± 0.14^e^	0.93	30.00 ± 0.0
5	30	36.66 ± 3.3	1.00 ± 0.00^f^	0.37	20.00 ± 0.0
15	5	67.77 ± 1.1	1.19 ± 0.09^f^	0.81	24.81 ± 2.6
15	15	72.59 ± 2.5	1.00 ± 0.00^f^	0.73	41.48 ± 1.4
15	30	33.33 ± 3.3	–		33.33 ± 3.3
30	5	30.00 ± 0.0	1.00 ± 0.00^f^	0.30	–
30	15	23.33 ± 3.3	1.20 ± 0.11^f^	0.28	10.00 ± 0.0
30	30	20.00 ± 0.0	–		20.00 ± 0.0

*Values are the mean ± SE. Means followed by the same letter in the column are not significantly different as indicated by Fisher's LSD (*P* = 0.05).

Well-developed rooted plantlets formed either from direct shoot production from axillary buds or indirectly from PLBs in the induction medium were planted out in pots containing one of six compost recipes and grown on in a glasshouse for 90 days. Some compost mixes were more successful than others. Out of the 360 rooted plantlets transferred in each experiment (Fig. 1E), the highest survival (68 %) was obtained in substratum containing crushed brick and charcoal, and shredded bark (1 : 1 : 1) with a layer of moss. Almost as successful, giving 63 % survival, was a mix of crushed brick and charcoal with decaying litter (1 : 1 : 1) plus a layer of moss (Table 3) (Fig. 1F).

**Table 3:** Re-establishment of D. longicornu plantlets after 90 days of hardening under the greenhouse conditions.

Treatments	Survival (%)	Height (cm)
Brick + charcoal (1 : 1)	21 ± 1.4	3.44 ± 0.20
Brick + charcoal (1 : 1) + layer of moss	38 ± 2.8	3.55 ± 0.25
Brick + charcoal + decaying litter (1 : 1 : 1)	32 ± 2.0	3.30 ± 0.11
Brick + charcoal + decaying litter (1 : 1 : 1) + layer of moss	63 ± 4.2	4.10 ± 0.30
Brick + charcoal + bark (1 : 1 : 1)	35 ± 1.4	3.05 ± 0.25
Brick + charcoal + bark (1 : 1 : 1) + layer of moss	68 ± 2.8	4.00 ± 0.40

Values represent the means ± SE.

Means of 60 plantlets per substrate were taken and the experiments were repeated three times.

## Discussion

The supply of growth regulators promoted the production of shoots from the axillary buds of nodal explants cultured on MS medium. This is probably an outcome of the habituated nature and juvenility of axillary buds. The juvenility of tissues is thought to be an important factor controlling cell proliferation in several orchids (Vij and Pathak 1989; Arditti and Ernst 1993; Vij *et al*. 1997). The axillary buds of *D. longicornu* explants responded differently to the two auxins NAA and 2,4-D. Shoot buds and PLBs were seen emerging from the explants given 2,4-D. Such a varying response in the form of shoot buds/PLBs has been attributed to the genetic and/or source-related physiological intricacies (Vij *et al*. 2000). In *D. longicornu*, the maximum number of shoots generated from each explant was recorded in medium supplemented with 30 µM NAA. Vij and Kaur (1998) also reported similar results where NAA-enriched medium favoured multiple shoot bud formation in *Malaxis acuminata*. Similarly, there are earlier reports on accentuated regeneration potential of *Dendrobium moschatum* pseudobulb explants (Vij and Sood 1982). In the present study, the regeneration pathway to shoot formation was directly through shoot bud formation and also through PLBs. When present, PLB formation was mostly restricted to media containing 2,4-D. The suitability of 2,4-D for both callusing and PLB formation in the case of *Dendrobium* has been reported previously (Kanjilal *et al*. 1999; Nasiruddin *et al*. 2003; Das *et al.* 2008). On the other hand, the incorporation of BAP in the medium promoted only the formation of shoot buds not PLBs. The activation of meristematic activity in explants by BAP has been found by others to be obligatory for the development of multiple shoot buds (Vij *et al*. 2000; Kosir *et al*. 2004).

In the present study, the response of explants to BAP in combination with auxins (NAA and 2,4-D) differed according to concentration. Tokuhara and Mii (1993) reported that the combination of hormones was of key importance for the micropropagation of *Phalaenopsis*. A stimulatory effect of BAP and NAA together in the medium has been reported for certain species of orchids before (Kosir *et al*. 2004). While some authors have reported reduced induction and regeneration in medium supplemented with NAA (Arditti and Ernst 1993), others reported that an appropriate combination of NAA and BAP stimulated shoot formation (Tokuhara and Mii 1993; Tisserat and Jones 1999; Roy and Banerjee 2003). Similar results were also obtained in our study wherein a maximum number of shoots and BFC were recorded in medium containing a combination of BAP (5 µM) and NAA (15 µM). The interactions of BAP with 2,4-D induced both PLBs and shoot buds from cultured axillary buds. Kusumoto (1979) had reported multiple PLB formation in *Cattleya* with kinetin (KN)/BAP in combination with 2,4-D. Although some authors reported the induction of shoot buds without the intervention of callus and PLBs (Devi *et al.* 1998), others have reported the formation of PLBs from different explants (Kim *et al*. 1970; Sharon and Vasundhara 1990). Since many orchid species require auxins and/or cytokinins for shoot and PLB formation (Arditti and Ernst 1993), the combination, concentrations and the ratio between them are critically important. In the present study, the explant response in terms of PLB induction was highest in the medium supplemented with 2,4-D or NAA in combination with BAP at the same concentrations (15 μM). The response of the explants to PLB formation varies from species to species and from explant to explant used (Teng *et al*. 1997). A high ratio (12.2) of NAA to BAP in *Spathoglottis plicata* was reported to be best for the induction of PLBs from nodal explants (Teng *et al*. 1997). However, a low ratio of NAA to BAP, 0.12 in the case of *Phalaenopsis amabilis* (Tanaka and Sakanishi 1985) and 0.42 in *Dendrobium antennatum*, was reported to be suitable (Kukulczanka and Wojciechowska 1983). Also, in several hybrid species of *Aranda*, a ratio of 1.23 for NAA to BAP has been found to be most effective (Khaw *et al*. 1978).

The survivability of the micropropagated plantlets on being transferred to pots depends on their proper acclimatization. *Dendrobium longicornu* is epiphytic in nature and the substratum should reflect this by combining water-holding capacity with good drainage. Crushed charcoal and brick pieces were therefore adopted as the basis for the six compost mixes we tested. The highest survival levels (over 60 %) were achieved when this combination was augmented with shredded bark or decaying litter plus a covering layer of moss for water retention (Dohling *et al*. 2008).

## Conclusions and forward look

*In vitro* multiplication of orchids makes an effective contribution to saving rare species from extinction. This is the first report of a successful and efficient protocol for *in vitro* propagation of the threatened medicinally useful epiphytic orchid *D. longicornu*. The method uses nodal explants with an axillary bud cultured *in vitro* on MS semi-solid medium supplemented with the cytokinin BAP and the auxin 2,4-D, each at 15 μM. Regeneration of viable rooted shoots is mediated by combination of direct shoot bud formation and indirectly via PLBs. The technique is likely to be widely applicable but the growth regulator component may need adjustment depending on the species, and the physiological state and nutrient environment of the source material.

## Sources of funding

Financial support received from the Department of Biotechnology, Government of India vide grant No. BT/PR2321/SPD/11/199/2001 is gratefully acknowledged.

## Contributions by the authors

All the authors contributed to the same extent overall and have seen and agreed to the submitted manuscript.

## Conflicts of interest statement

None declared.
